# Being healthy: A Grounded Theory study of help seeking behaviour among Chinese elders living in the UK

**DOI:** 10.3402/qhw.v9.24820

**Published:** 2014-10-21

**Authors:** Zhenmi Liu, Kinta Beaver, Shaun Speed

**Affiliations:** 1 School of Nursing, Midwifery and Social Work, University of Manchester, Manchester, UK; 2 School of Health, University of Central Lancashire, Preston, UK

**Keywords:** help seeking, elderly Chinese, grounded theory

## Abstract

The health of older people is a priority in many countries as the world's population ages. Attitudes towards help seeking behaviours in older people remain a largely unexplored field of research. This is particularly true for older minority groups where the place that they have migrated to presents both cultural and structural challenges. The UK, like other countries, has an increasingly aging Chinese population about who relatively little is known. This study used a qualitative grounded theory design following the approach of Glaser (1978). Qualitative data were collected using semi-structured interviews with 33 Chinese elders who were aged between 60 and 84, using purposive and theoretical sampling approaches. Data were analysed using the constant comparative method until data saturation occurred and a substantive theory was generated. “Being healthy” (the core category) with four interrelated categories: self-management, normalizing/minimizing, access to health services, and being cured form the theory. The theory was generated around the core explanations provided by participants and Chinese elders’ concerns about health issues they face in their daily life. We also present data about how they direct their health-related activities towards meeting their physical and psychological goals of being healthy. Their differential understanding of diseases and a lack of information about health services were potent predictors of non–help seeking and “self” rather than medical management of their illnesses. This study highlights the need for intervention and health support for Chinese elders.

Appropriate and timely help seeking behaviour can solve, prevent, or at least ameliorate many health problems. However, delays in help seeking behaviour are frequently seen among people with certain chronic diseases. These delays are more common among older patients and particularly so for those from black and minority ethnic (BME) groups (Bett, Tonkin, Thompson, & Aroney, 2005; McGinn et al., 2005; WHO, 2010). A delay in help seeking can bring fatal consequences and is a major problem for both patients and health care providers (WHO, 2010). The rates of chronic diseases, such as cardiovascular diseases, are much higher in the older population who attend late at services because they accept symptoms and present only when a new emergency or health care problem occurs, if they attend at all (Goldberg et al., 1998; Howse, Ebrahim, & Gooberman-Hill, 2005; King, Sanguins, Mcgregor, & Leblanc, 2007; Tod, Read, Lacey, & Abbott, 2001). Additionally for minority groups, cultural and ethnic issues relating to place and health care systems also play an important part in delayed help seeking (Marmot, 2010; Scheppers, Dongen, Dekker, Geertzen, & Dekker, 2006). There is a paucity of information about the specific social processes for help seeking among minority ethnic groups and how the interactions affect the services people from minority groups receive. This information could be invaluable to the provision of culturally sensitive services to older people from minority groups.

The Chinese ethnic population are among the UK's largest visible minority groups. The Census data indicate that the Chinese ethnic population represents 0.7% of the UK population in 2011 with approximately 5% of the total number of Chinese immigrants being aged 65 years and over (Office for National Statistics, 2002, 2012). However, this group of people are noted to be consistently unreported in Census data and local area population statistics, meaning that there is a considerable “hidden population” of mainly older Chinese people in the UK (Gao, 2010). Most elderly Chinese immigrants are first generation and tend not to speak English as a first language (Sproston, Pitson, Whitfield, & Walker, 1999).

A limited academic international literature suggests that Chinese immigrants are known to have an independent and unique cultural outlook which influences almost every aspect of their life (Chung et al., 2012; Kwok & Sullivan, 2007). Chinese elders are known to rely on traditional eastern values especially when considering issues related to health, including their approach and uptake of exercise, and their beliefs about the restorative and curative nature of diet and about the importance of rest, relaxation, and balance in their lives (Chen, 2001; Green, Bradby, Chan, & Lee, 2006; Hsu-Hage et al., 2001; Huang & Spurgeon, 2006; Kwok & Sullivan, 2007; Ma, 1999; Pang, Jordan-Marsh, Silverstein, & Cody, 2003; Wong & Pang, 2000). This can lead to a distinctive approach to health-related help seeking behaviour different to indigenous or other minority groups within a particular country. This study aims to make both an empirical and theoretical contribution to understanding by exploring the health-related behaviours of Chinese elders in their daily life, particularly help seeking behaviour when facing health problems.

## Methods

A qualitative approach using classic Glaserian grounded theory was chosen to gain insight into the experiences and the main concerns of the participants leading to the development of a theory. An important principle was that the researcher began the project without a preconceived theoretical perspective but with an area of interest. In doing so, the grounded theory is developed during the process of investigation, rather than preconceived (Glaser, 1978, 1992; Glaser & Strauss, 1967). The key feature of this study was the emergence of both the research problem and research questions. In this way, it is suggested that the original aims may be modified in order to investigate more fully what was actually happening within the social sphere. This study developed an understanding of the concerns of Chinese elders and the interview guide was continually modified and finally focused on the general health behaviour process, including the management of health-related events in daily life including help seeking behaviour when facing health care problems. As the research progressed these aims became the main focus of the study since data analysis revealed they were central to the emerging theory. The initial aim of this study was to investigate the barriers to help seeking for older Chinese people' this original aim then developed into the final aim as discovering the basic social processes involved in accessing health care services for this group of people.

### Sample selection and recruitment

A theoretical sampling approach with an initial purposeful strategy, suggested by Glaser (1978, 1992), was adopted to guide the selection of the potential participants in this grounded theory study. As little was known about the help seeking behaviour among the elderly Chinese ethnic group, this process allowed recruitment to be initially based on the information from the pre-field work literature review. The target population were first generation Chinese elders living in the UK, healthy (self-defined as elders) and aged 60 years old or over.

Based on the principles of maximum variation in sampling (Glaser, 1978, 1992; Glaser & Strauss, 1967), the initial purposive sampling aimed to recruit elders from diverse backgrounds. The study sites were large Chinese social clubs, including an Elderly Luncheon Club, a Christian club, and a Buddhist temple in the North West of England. Through this approach, participants with diverse backgrounds were engaged in this study. Movement between the sites came from recommendations of participants at earlier sites and is a key aspect of purposive sampling (Coyne, 1997; Glaser, 1992). After initially simultaneous data collection and analysis, theoretical sampling (Glaser, 1992) was used to conduct recruitment in order to generate theory.

All of the participants were recruited from clubs for older Chinese peoples which were social and community organizations rather than health care services. This was an important aspect of the recruitment process as we wanted to recruit participants who self-identified as being healthy.

### Data collection

Semi-structured interviews with a Glaserian approach and open-ended questions were used in this exploratory study, particularly in the purposive sampling phase of the investigation. The interviews were designed to collect qualitative data regarding the cultural and basic social context of Chinese elders’ lives in the UK in relation to their health and help seeking behaviour. The questions about the key themes or concepts, such as health experiences and management of health problems, were asked of those involved. Furthermore, the kind and form of questions went through a process of development to ensure the topic and focus of the interview was developed alongside the analytical process. In this way, deep and rich information was obtained. The questions became more focused and evolved as the analysis progressed and the categories emerged through the analytic and reflective process. In the spirit of grounded theory, the reflexivity of the research team was essential in guiding the progress of the study and added to the rigour of the approach. The interviews were conducted by the main author who is an experienced qualitative researcher. She is a Chinese national who is fluent in Mandarin, Cantonese, and English. Interviews were conducted in the participants’ language of choice.

In the initial stages of the investigation during the first few interviews, the broad questions, such as “how about your health recently?”, “tell me about your health in general,” were discussed. This enabled the participants to realize that there were no right or wrong answers only their opinions and ideas. The help seeking behaviours were explored in-depth and focused on their individual experiences and social processes to provide us with a chance to explore the minutiae of the participants’ perceptions about their own health. Further on in the interview, participants were asked about their normal health seeking pathways: “Can you give me an example of a health problem you have had in the past and what did you do about this?” These questions elicited information about health seeking behaviour.

During the next phase of the study as the process of data collection proceeded, the topics in the interview guide narrowed down to certain themes in order to gain a rich and dense category and thus a solid theory. Theoretical sampling at this point included a detailed exploration of the emerging themes and included issues relating the level of help seeking and the basic social processes involved in this. Key informants were recruited, such as those who had had an experience of illness and help seeking but who still self-identified as being healthy. Participants were recruited from a core group of people who had agreed to take part and who were judged as being able to contribute to the developing theory.

Finally during interviews at the theoretical sampling stage, the interview questions aimed to expand on these categories, such as “being a healthy person” and “cure (symptoms) model versus treat (root causes) model,” by asking, for example, “Could you say more about how you see being cured?” in an open-ended manner. The category “cure (symptoms) model versus treat (root causes) model” was enriched most during this theoretical sampling phase and will be used as an example. When this category emerged, it was lacking in density since elders did not explain or ask for more from doctors if they thought doctors did not provide what they wanted. Thus, the directions of theoretical sampling were focused on “What do chronic diseases mean to the patients?/What do they know about these diseases?” and “Where does the information come from (if it does not come from doctors, why not)?” After exploring these issues in-depth, this category was renamed “being cured” with dense properties and the internal connections, such as “ineffective culture-related communication” and “avoiding bothering doctors unnecessarily.”

### Data analysis

All interviews were conducted in the participants preferred language (Mandarin or Cantonese) and recorded following consent from participants. Back translation was conducted and the similarities and differences were compared for accuracy in English version.

Data analysis was initiated as soon as the first interview was completed; a two-stage coding process was applied, starting with a substantive coding (open and descriptive codes) followed with theoretical coding (increasingly higher-level conceptual categories that encompassed the descriptive codes and the relationships between them). The grounded theory concepts of constant comparison method and theoretical sensitivity were used during the whole study process to ensure that the developing codes and theories remained grounded in the data. The emerging ideas from the initial analysis were compared with previous and subsequent interviews, and the ideas generated were noted in the form of theoretical memos using constant comparison. Thus, the subsequent interview guides were modified and based on the analysis of previous interviews in order to expand the codes and theories in the setting. In the study reported here, it enabled the identification of consensus among participants as well as disagreements or differences of opinion with the analysed data. The constant comparison of the emerging ideas meant that interviews were often re-analysed in the light of new data using constant comparison as a method of investigation and inspection of the data. This process also helped to ensure that the analysis process was grounded in the data and the interview schedule.

### Ethical considerations

The study received ethical approval from the Research Ethics Committee at the University of Manchester, as being in accordance with ethical standards. Participants were given verbal and written information about the study and flyers with contact information in case they felt the need further contact. All participants gave written informed consent.

### Academic rigour

Following these generally accepted measures which were conceptualized by Lincoln and Guba (1985), and modified later by Denzin and Lincoln (2000), credibility, transferability, dependability and confirmability were taken to ensure the rigour of this qualitative research. Throughout the interviews, especially the first few purposive interviews, the interview guide was the main tool to guide the conversation. However, some ideas or topics emerging from the previous interviews were added to the subsequent interviews to gain validation among later participants. Additionally, preliminary findings were shared with participants during second interviews that took place later in the research process. In the theoretical sampling phase, summaries of the current findings, including themes, were presented to participants for comments and evaluations of credibility for the participants. In terms of transferability, a thick description of the data as well as the context and the interactions between the participants and the researchers were presented. Detailed quotes and often more than one exemplar are offered in the findings section so that readers can make their own assumptions from the data and the exemplars presented. The data were also compared with the relevant literature, and there were some aspects which clearly resonated with overseas Chinese immigrants in other localities across the world. This provided some confirmation that the findings were potentially transferable to the real life of Chinese immigrants living in other societies. Dependability and confirmability are the assessments of the quality of the integrated processes of data collection, data analysis, and theory generation to measure how well the inquiry's findings are supported by the data collected (Lincoln & Guba, 1985). An audit trail consisting of the original transcripts and data analysis documents trail was kept. Thus, the dependability and confirmability of the project, as well as the completeness and availability of auditable documents, were assessed by the experienced research team.

In terms of the theory of being healthy, Glaser suggests that we consider fit, work, relevance, and modifiability (Glaser and Strauss, 1967). In the theory presented here, we are confident that the efforts we took to ensure that the analysis process which included rigorous open coding, comparative analysis, memoing, theoretical sampling, and selective coding have provided a guarantee of fit. A theory works when it is helpful in explaining and phenomenon (Glaser and Strauss, 1967). This thesis presented in this paper has led to a greater level of understanding of Chinese elders’ behaviour in their daily life based around their perception of being healthy and their basic social processes surrounding health-related help seeking. According to Glaser and Strauss (1967) if a theory is to work, then it should be relevant and contextualized, and the theory presented here situates and explains Chinese elders’ perceptions of health within a context that relates to their help seeking behaviour. Modifiability is the way in which a theory can be adapted and expanded within similar or different contexts (Glaser and Strauss, 1967). We are confident that the core category of being healthy, for example, can explain social processes in other parts of the world where Chinese people live. We are also confident that the barriers to help seeking will be different depending on the context and the health care system. This suggests that our theory may be stable and explanatory enough but may also be modifiable to other contexts, which is a key aim of grounded theory which aims to generate theory to be tested further.

## Findings

Thirty-three first generation Chinese elders (60–84 years, mean 71 years) from the North West of England participated directly in the study (Table I). More than two-thirds (23/33) were female. Approximately, two-thirds (22/33) had the equivalent of primary school education or was uneducated. None of the participants could communicate well in English. Participants had lived in the UK from 1.5 to 54 years (mean 25 years). It might be expected that they were confident and competent users of the UK health care services but this was not the case.

**Table I:** Participant characteristics.

Gender	
Male	10
Female	23
Age	
60–69	12
70–79	18
80–84	3
Years of residence in the United Kingdom	
1.5–20	16
20–39	7
40–54	10
Education level	
None or Elementary school	22
High school level or higher	11

Table II provides an overview of the results as discussed below.

**Table II:** Core Category and four sub-categories.

Core category=being healthy as a pervasive goal			

Category 1	Category 2	Category 3	Category 4
			
Self-management	Normalizing/minimizing to be healthy	Access health service	Being cured
• Personalized self-care and self-treatment	• Creating a sense of normality • Knowledge about health issues • Old age and accepting—emotional stoicism and fatalism	• Roles of family • Controversial health care service in the UK	• A dual character of Western medicine • Variation in appraisal towards practitioners • Avoiding bothering doctors unnecessarily • Passive dependency upon doctors

### Being healthy

Being healthy was the main concern among the participants because the ultimate aim of Chinese elders’ behaviours was to maintain health. Chinese elders’ health beliefs were found to be a set of principles and practices associated with nature, based on a holistic view, which included physical, psychological, and environmental considerations. The maintenance of health and the prevention of disease were the Chinese elders’ strongest motivation. They emphasized their strict management of their daily healthy routines, including diet, sleep and rest, exercise, as well as physical and mental discipline.

What we can do is concerning the food, eat fresh, or natural food. Another thing is rest, have good rest as well as good sleep … We should keep balance, keep peace. Getting older, diseases are always there. There seems no method to stop it … Doing exercise is always important … When I feel uncomfortable, such as dizzy … I guess maybe because of my blood pressure, I will adjust my diet, eating light foods.

When facing health problems, along with adjustments to lifestyle, maintaining a stable psychological state and avoiding emotional fluctuations were the key factors to re-establishing their health.

I began to know that mood affects health a lot, and is really a big problem … I felt that I have to avoid being angry or listening to bad things. … I shall just walk away and not hear it … In this way I get healthier.

The key point in these excerpts is that elders undertake this activity through self-assessment and self-management that does not usually involve health care professionals and is not dependent on the medical resources around them.

### Self-management

Being healthy involved a significant degree of self-management. Elders took personal responsibility for activities and strategies which had the intention of maintaining or promoting health. Traditional Chinese medicine (TCM) and other non-prescribed health care products, which were considered as having few or no side effects when compared with western medicines (WMs), were commonly used as an effective way to self-manage. It is clear here that the services on offer within the Western sphere of medicine were deemed to be inadequate.

Generally, if my blood pressure is not very high, I won't have pills (WM medication for hypertension). About 140, 150, it is not very high, why should I continue taking pills? I think if I keep having medicine, one day when I really get ill and need medicine, there will no effect of the pills. Actually I consider my health a lot during the daily life … There are also some health-protective remedies, which were brought from China … Seems good at lowering blood sugar and blood pressure …

Older peoples’ self-confident or assertive attitudes to self-management made them rely and trust these alternative medicines but overlook the functions of the prescribed WM. This was sometimes quite dangerous as the relief of some symptoms could conceal some potential diseases; and the concept *no symptom, no disease* made elders reluctant to seek help. It is notable that WM approaches and services figured rarely in their accounts of self-management as they avoided the services that were on offer to them.

### Normalizing/minimizing to be healthy

Part of the desire for being healthy was seen in the way that most elders reported experiencing an illness, sometimes a major and ongoing problem, but almost all evaluated themselves as “normal” by denying negative disease-related events and symptoms. They believed that avoiding anxiety, particularly anxiety related to illnesses and keeping peace, was helpful for their health. The term *minor* was commonly used to create a feeling of being healthy. Often they provided other physiological explanations for the signs and symptoms of ongoing health problems in particular the natural process of aging rather than a health problem that can be ameliorated or at least managed.

In fact I feel uncomfortable sometimes … Anyway, I am so old, it good enough to be like this

This ultimately gave them a sense of comfort and led to self-management rather than help seeking or consultation with health services. Symptoms were ignored or managed rather than reported. Moreover, some elders knew that certain acute symptoms were associated with heart problems, such as *myocardial infarction (MI)* and *angina*; meanwhile, many used the term *sudden* to show their astonishment and helplessness, suggesting that heart problems were *unpredictable* and out of their control.

I know about this information (about heart) … I will pay much attention to it because I fear that one day I may fall asleep and suddenly cannot wake up, dead. I am very worried about it; because I am likely to lose my breath often during sleep … MI, I know it.

These fearful perceptions lead them to avoid thinking of illnesses, particularly coronary heart disease. With more investigation, it was discovered that many elders did not possess any actual knowledge of diseases and those medical terms they used. This process made a considerable contribution to a lack of help seeking. In sum, minimizing health related problems they experienced and involved made Chinese elders downplay the severity of symptoms or diseases and also the impact these aliments had on their lives. This meant that they would avoid help seeking unless absolutely necessary as help seeking may confront their process of minimizing and their desire to stay healthy and remain anxiety free.

### Barriers to access to health services

The role of family was reported as both promoting and hindering the help seeking process. The family is the primary source of assistance for elders in difficulty or in need. The support from their family included transportation, translation, and information support. However, the family sometimes also hindered elders’ help seeking.

I have not mentioned this heart problem to the doctor here. My son told me if there is no symptom of my heart, the doctor won't check it for me. I had headache several times; my chest felt uncomfortable and I can't breathe well … my son said it can only be checked out when I have symptom …

This situation occurred frequently among elders who were experiencing chronic ailments. It was further noted that elders preferred to choose to follow their children's suggestion of not seeking help. Meanwhile, as elders used self-management and normalizing strategies, they became more convinced of the efficacy and importance of their self-management techniques, and hence this strengthened their conviction to avoid seeking further medical help. This had obvious consequences for the late presentation and acute presentation at health care services. In addition, other institutional factors such as long waiting times and the referral system also influenced elders’ help seeking decisions.

You have to go to the GP first. If you have an illness, maybe a big illness, you still have to wait the referral. It's quite troublesome … Sometimes the symptoms turn better and the appointment still does not arrive … just cancel it or if it gets worse I will go to A and E (accident and emergency)

It is important to recognize this stage in the help seeking process as the elders in this study suggest that help seeking is a last resort in a long process and occurred when their own self-management strategies had failed. The data indicate that there are internal factors including their assessment of their own health, family dynamics, and characteristics of the health services they wish to approach that become barriers to accessing health service.

### Being cured

The term “cured” was a direct translation from Chinese which meant that symptoms were relieved without eradication of the “root cause” of the illness and was one of the major considerations for elders in making decisions about seeking help.

I feel the doctor don't do well on telling patients how to take care of themselves and how to promote health … busy … They are in a hurry, and want to finish treating you quickly … If I ask, doctor will somehow answer … just several words … I think they should concern themselves with what patients want … lack of information of the disease. That's a big problem.

Lack of “understandable” health-related information from doctors also led to dissatisfaction and distrust as they felt ill informed and unable to understand their treatment adequately. Furthermore, this discontent was also pronounced with regard to WMs prescribed by professionals. The strength and invasive nature of prescriptions made elders feel anxious about long-term medications. They suggested that when compared with the situation in China where doctors prescribed TCM as a way to treat the side effects of WM, elders showed a negative view towards the British WM professionals and those medicines.

I have diabetes and high blood pressure … GP prescribed some medicine for me to control them. Only TCM can cure the source of illness like peeing too much. WM can only control the symptom and how about the side effects? There is nothing to balance the bad effects.

They believed that they were able to gain treatment for both symptoms and the root cause of illness via TCM, while the function of WM was considered as symptom in remission only. Given that most of the services around them were geared towards the Western approach, it was clear that there would be problems with access as a consequence of this.

Recognizing that symptoms are only cured and the root cause is not eradicated meant that elders were reluctant to seek further help. Some, therefore, made a decision not to bother and sought a cure elsewhere:

I feel the doctor here don't do well on telling patients how to take care of themselves and how to promote health, they don't talk much with patients, this is no good … I think it is better not to trouble doctors as much as we can.

And

When getting older, people should understand the importance of health promotion … everyone knows his or her own body than anyone else, not doctor.

It is clear from the latter quote that the participants were reluctant to seek help and preferred to develop self-management strategies.

## Discussion

**Figure 1:**
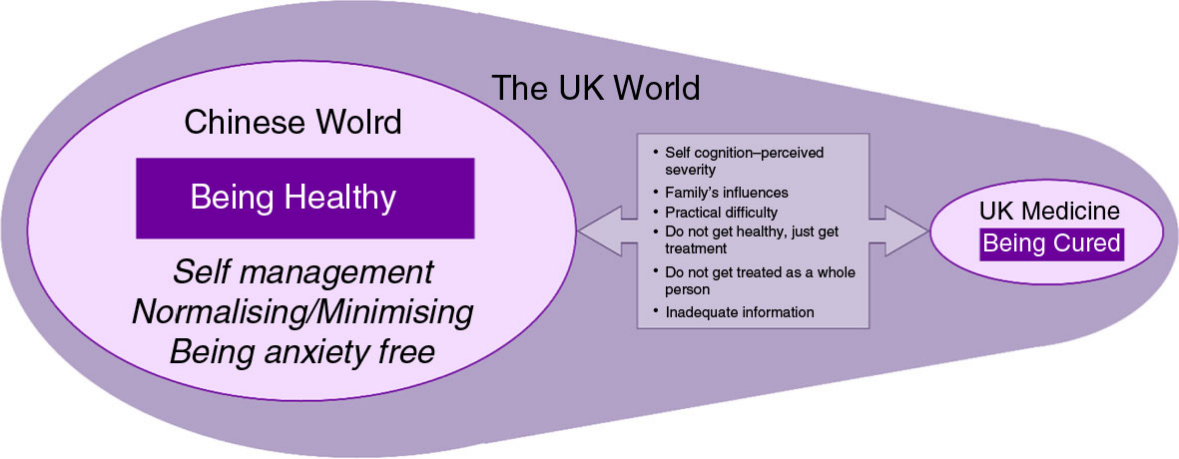
Dynamic process of Chinese elder's help seeking.

Chinese elders’ help seeking behaviour was mainly a form of self-management and non–help seeking until “necessary” (“being out of control” or “beyond self-management” from the elders’ perceptions). The nature of the relationships between the elements of the theory of being healthy were conceived as dynamic, cyclical, and interconnected. This is shown as a theoretical model in Figure 1.

A secondary literature review, self-management as a concept has been reported by a small number of other researchers as one major approach utilized by Chinese elders when dealing with health problems. This has been found in countries such as America (Ma, 1999; Pang et al., 2003), Canada (King et al., 2007), and Australia (Hsu-Hage et al., 2001; Kwok & Sullivan, 2007). In the main, these studies have reported the end stage or the results of self-management among Chinese people and recorded that they underutilized health care services. Less attention has been given to why this happens, rather just that it does. Thus, it has been suggested that Chinese people have relatively less check-ups or professional care when feeling ill compared with the local population (Aroian, Wu, & Tran, 2005; Hsu-Hage et al., 2001). The general ideas from the literature are that Chinese people present late, usually with severe illness, and require costly interventions.

Some evidence within the UK has suggested that Chinese older people self-report good health (Office for National Statistics, 2002, 2012; Sproston & Mindell, 2006). This concurs with our findings here where the participants in this study were ever keen to report good health as a consequence of self-management even when they had considerable morbidity related to chronic illness. The previous evidence may be an artefact of the methodology (quantitative surveys for example) which elicit favourable responses rather than true reflections of the state of health. None of the identified studies commented on why Chinese people were extremely diligent in self-management and how they managed their health in a defined area where care is offered, which is the subject of this paper. This present study has added important evidence and insights for understanding Chinese people's initial and continued help seeking behaviour. It may be that these local findings have resonance for other geographical locations.

Among the practical barriers during help seeking, a more frequent problem reported in the literature (Green et al., 2006; Kwok & Sullivan, 2007; Ma, 1999) was the communication barriers with WM staff. This was not only due to English language utilization but also to conceptual misunderstandings arising from cultural diversity which were echoed in the findings of this study. Further support for these findings was provided by only two studies conducted among Australian Chinese people (Hsu-Hage et al., 2001; Tang & Easthope, 2000), and more research is needed in this area. “Being cured” in this present study illustrated this mismatch of expectations between Chinese patients and WM practitioners where their two worlds collide. An Australian study (Tang & Easthope, 2000) explained that, on the one hand, Chinese patients criticized WM doctors and nurses for not treating the roots of the disease, and on the other hand, WM professionals had difficulty in understanding what Chinese patients’ phrase the root cause meant as used in Chinese culture. This resulted in a decreased level of trust in WM professionals and this acted as a barrier for the use of health care service.

It is clear that despite a long and continued existence in many countries around the world, older Chinese people continue to hold onto their own perceptions of health. Despite many years of living and working in a host Nation such as the UK, both interpersonal and systemic barriers hinder help seeking behaviour and access to treatment for this increasingly aging sector of the population. Whilst these data suggest that this is true for the UK, we would propose that these findings may have resonance elsewhere in the world where similar patterns of migration have occurred. Chinese people have maintained a unique and sustained approach to health and health-related help seeking.

There are limitations to this study. The sample studied in this area was largely from one city in the North West of the UK. However, the population of Chinese people in this geographical area are similar to those migrant populations elsewhere in the developed world for example the United States and Canada (Kwok & Sullivan, 2007; Lai & Chau, 2007; Pang et al., 2003), which may mean that these data could be transferable to other migrant, first generation Chinese populations. As with most qualitative research the richness of the data sought in this naturalistic enquiry has meant that the sample is relatively small even if care was taken to ensure rigour in the sampling process.

The study has described a care trajectory of how older Chinese people have adapted (or not) their traditional beliefs and cultural practices to the health care system in the UK. This is the first time that a theory accounting for how Chinese elders manage their health events themselves and how they interact with others in a host society, including nurses and doctors when the health problems progressed to a perceived serious level. As evident from the literature, other studies to date have described the consequences of not seeking help. No previous studies have, to our knowledge, investigated how self-management influenced their initial help seeking behaviour or reported the implications of this in hindering the help seeking process. There is a need to address more closely the everyday processes and patterns of how Chinese elders are thinking and behaving and therefore the findings of this study may have the potential to inform and enhance the health care support received by the Chinese community where ever they reside.

Perhaps the most important message from this study is that if Chinese elders present for health-related problems at health care services, it is usually the end point in a complex process of self-management and general reluctance or avoidance of the health care system. Medical practitioners should be aware that this is a direct and serious request for help when their usual approaches to managing health have failed. Practitioners should be aware of the importance of this contact in their consultations with elders.
